# Asymmetric and Eccentric Laser Pointer Maculopathy in a Teenager

**DOI:** 10.18502/jovr.v20.17828

**Published:** 2025-06-13

**Authors:** Maria Krisch, Jordan Ueberroth, Mark P. Breazzano

**Affiliations:** ^1^Norton College of Medicine, SUNY Upstate Medical University, Syracuse, NY; ^2^Department of Ophthalmology & Visual Sciences, SUNY Upstate Medical University, Syracuse, NY; ^3^Flaum Eye Institute, Department of Ophthalmology, University of Rochester, University of Rochester Medical Center, Rochester, NY

##  PRESENTATION

**Figure 1:**
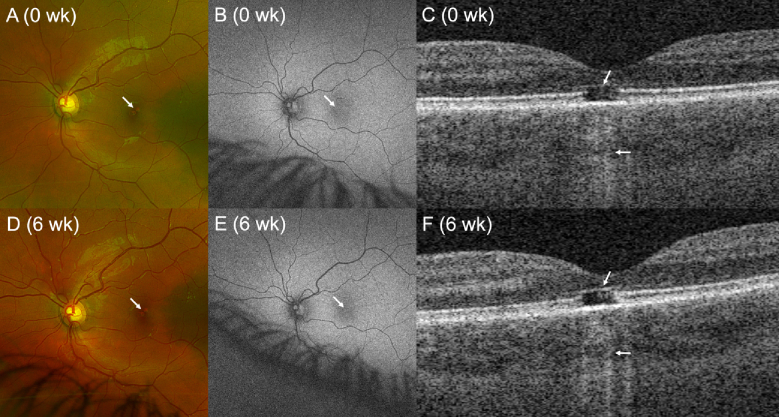
Multimodal imaging of laser pointer maculopathy in the left eye. (A) Ultra-widefield fundus photography from the initial encounter demonstrates foveal disruption (arrow). (B) FAF shows colocalizing hypo-autofluorescence (arrow). (C) Swept-source OCT demonstrates foveal ellipsoid zone disruption (top arrow) with choroidal hyper-transmission (bottom arrow), consistent with RPE/Bruch membrane complex damage. Central foveolar thickness is 236 microns. (D & E) Fundus photography and (E) FAF at six-week follow-up reveal stable foveal disruption (arrows). (F) OCT demonstrates subtle improvement in the sub-foveal disruption (top arrow) with residual choroidal hyper-transmission (bottom arrow), representing persistent RPE/Bruch membrane damage. Central foveolar thickness is unchanged. FAF, fundus autofluorescence; OCT, optical coherence tomography; RPE, retinal pigment epithelium.

A previously healthy 13-year-old boy presented with a central “blind spot” in his left eye lasting three months. At presentation, left eye Snellen acuity was 20/40 with an abnormal foveal light reflex noted on examination. Ultra-widefield fundus imaging revealed foveal disruption [Figure 1A], while fundus autofluorescence (FAF) demonstrated a corresponding area of hypo-autofluorescence in the fovea[Figure 1B]. Swept-source optical coherence tomography (OCT) of the macula revealed a sub-foveal optical gap with outer retinal disruption and hyper-transmission into the choroid, consistent with damage to the retinal pigment epithelium and Bruch's membrane [Figure 1C].

**Figure 2:**
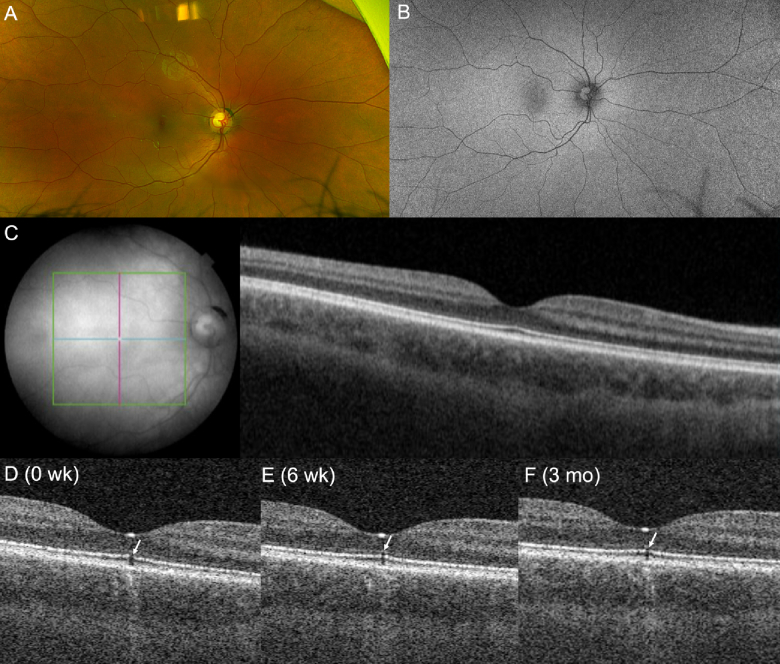
Multimodal imaging of the right eye with subclinical laser damage. (A) Ultra-widefield fundus photography and (B) FAF on initial encounter demonstrate “normal” findings. (C) The default, central B-scan with swept-source OCT through the fovea on initial encounter is “normal.” (D) A single, eccentric OCT B-scan, adjacent to the default central scan, demonstrates a small sub-foveal optical gap (arrow). (E) OCT at six-week follow-up demonstrates an improved but persistent sub-foveal optical gap (arrow). (F) OCT at three-month follow-up demonstrates persistence of this initially improved sub-foveal gap (arrow). FAF, fundus autofluorescence; OCT, optical coherence tomography.

The right eye was asymptomatic with 20/20 Snellen acuity. Initial imaging of the right eye, including en face macula thickness map, default central B-scan with OCT, fundus imaging, and FAF, appeared normal [Figures 2A, 2B, & 2C]; however, a small sub-foveal optical gap was detected on a single, eccentric B-scan within 3D OCT cube, consistent with subclinical damage [Figure 2D].

Ultimately, the patient admitted to staring into a green laser pointer for an unknown duration, supporting a laser-induced maculopathy. At six-week follow-up, the patient's left eye visual acuity remained stable at 20/40, with a stable central scotoma and abnormal macular light reflex. Imaging at this time showed persistent foveal disruption in the left eye on fundus photography [Figures 1D & 1E], while the optical gap exhibited subtle improvement on OCT [Figure 1F]. The right eye's optical gap on OCT persisted, though mildly reduced in size, at the six-week [Figure 2E]and three-month [Figure 2F]follow-up visits.

##  DISCUSSION

This case underscores the importance of thorough ocular examinations and reviewing all imaging components when diagnosing laser-induced maculopathy. In this case, the damage in the fellow eye, undetected in standard imaging, was revealed only by careful “scrolling” through the entire OCT macula cube. While laser-induced maculopathy typically affects only the exposed eye, the subclinical findings in this case suggest that all patients should undergo a thorough bilateral ophthalmic workup.

Laser-induced maculopathy can have lasting visual complications, disproportionately affecting children.^[1]^ The growing availability of unregulated, high-powered laser pointers has contributed to an increased incidence of this condition.^[2,3]^ Symptoms typically present as a sudden, painless vision loss following direct laser exposure.^[1]^ The degree of visual impairment can vary, with some patients experiencing mild improvement, while others endure long-term complications such as subretinal neovascularization or permanent vision loss. Diagnostic imaging, including color fundus photography and OCT, is essential for identifying laser-induced maculopathy. Often, OCT reveals macular plaques and hyper-transmission into the choroid.^[4]^

The prognosis in laser-induced maculopathy depends on the laser's wavelength, power, and the extent of retinal injury.^[1,5]^ The US Food and Drug Administration classifies lasers based on their potential for harm, with research indicating that higher-powered lasers result in more severe, immediate damage and worse visual outcomes.^[1,2,5]^ Despite regulatory efforts, many laser pointers on the market are mislabeled and more hazardous than consumers are led to believe.

Overall, this case underscores the importance of thorough bilateral evaluation in suspected laser maculopathy and emphasizes the need for heightened awareness regarding the risks of laser misuse.

##  Ethical Considerations

The need for patient consent was waived due to the minimal risk offered to patients and the retrospective nature of this case study. All procedures were reviewed and performed according to the tenets of the Declaration of Helsinki.

##  Financial Support and Sponsorship

None.

##  Conflicts of Interest

MPB reports personal fees from Iveric Bio/Astellas and ONL Therapeutics, unrelated to the present work. None of the other authors has any disclosures or commercial relationships to report.
